# Surface defect detection of industrial components based on vision

**DOI:** 10.1038/s41598-023-49359-9

**Published:** 2023-12-13

**Authors:** Zhendong Chen, Xuefeng Feng, Li Liu, Zhenhong Jia

**Affiliations:** 1 College of Information Science and Engineering, Xinjiang University, Urumqi, 830046 China; 2 Xinjiang University Signal Detection and Processing Autonomous Region Key Laboratory, Urumqi, 830046 China; 3 Xinjiang Uygur Autonomous Region Research Institute of Measurement and Testing, Urumqi, 830000 China

**Keywords:** Applied optics, Optical physics, Information theory and computation, Optical physics

## Abstract

Early and effective surface defect detection in industrial components can avoid the occurrence of serious safety hazards. Since most industrial component surfaces have tiny defects with high similarity to the detection background, there are often issues of missed or false detections when defects are detected, leading to low detection accuracy. To deal with the aforementioned issue, this essay suggests a high-precision detection model for surface defects in industrial components based on the YOLOv5 algorithm. First, the original spatial pyramid pooling (SPPF) is innovated by proposing the SPPFKCSPC module, which improves the network's capacity for feature extraction from targets at different scales and fuses multiscale features better. Then, C3 is combined with SPPFKCSPC and replaces the C3 module of the backbone network, which improves feature expression and enhances the receptive field of the network. Finally, the coordinate attention mechanism (CA) has been embedded into the YOLOv5 neck network, and the bounding box regression loss function of the algorithm is improved to EIOU, not only improving the precision of the target localization and recognition model but also enhancing the overall network performance. Based on the public datasets NEU-DET and PV-Multi-Defect, multiple sets of experiments were conducted using innovative algorithms. On the NEU-DET dataset, we got a mean average accuracy (mAP) of 88.3%, which is 7.2% greater than the original approach. On the PV-Multi-Defect dataset, the mAP value reached 97.5%, an improvement of 1.5%. As shown by the experimental data, the detection results significantly improved.

## Introduction

Traditional defect diagnosis uses manual visual inspection with low detection accuracy and efficiency. With the continuous improvement of technology, machine vision-based inspection and the deep learning-based inspection are the two main categories of defect detection techniques currently available. Machine vision detection refers to using machines to replace human eyes in various measurements and judgments. Compared with manual visual detection, machine vision detection greatly decreases labor and time costs, so it is widely used. Suvdaa et al.^1^ introduced a combined scale invariant feature transform (SIFT) and support vector machines (SVM) technique for surface defect identification, using SIFT to identify defects and extract features from the target and SVM to classify the retrieved features. Xiao-Cong^2^ proposed a new defect detection model to determine whether a defect exists or not, which classifies the defect image by hybrid support vector machine-quantum particle swarm optimization (SVM-QPSO) model for better detection of surface defects. In order to extract robust features, Gyimah et al.^3^ used a nonlocal (NL) technique with wavelet threshold filtering and completely local binary pattern (CLBP). These features were then fed into a classifier for surface defect detection. All of the above methods use traditional methods for feature extraction, and then machine learning is employed to categorize the features. However, because most of the defects have irregular distribution and inconspicuous texture, it is difficult to accurately extract the features only by traditional feature extraction algorithms, which leads to difficulties in using detection algorithms, and detection accuracy and efficiency are extremely low. Therefore, improving the feature extraction capability for defective images is critical.

To solve the problems in the above situation, deep learning techniques are gradually being used with defect detection^4^. The deep learning-based defect detection approach can use the defect image as the network input immediately and effortlessly retrieve the input image features. Since deep learning technology offers the benefits of high detection accuracy, quick recognition speed, and high adaptability compared to conventional methods, deep learning-based methods are often used to detect defective conditions in components in actual production. Single-stage and two-stage target identification algorithms based on deep learning are the most common. The mainstream two-stage algorithms are R-CNN, fast R-CNN, faster R-CNN, etc.^5–7^. The two-stage algorithm divides the generation and prediction of candidate frames into two stages. The detection accuracy is improved relative to nondeep-learning algorithms, but the model used for detection is more complex, resulting in longer inference times. To improve detection efficiency, single-stage detection models were proposed. Earlier single-stage detection algorithms include SSD^8^ and YOLO series networks^9–12^. Lv et al.^13^ proposed an efficient and accurate image defect detection model using the SSD algorithm as a framework for detecting and improving the backbone network as ResNet to increase the detection rate. Hatab et al.^14^ used the YOLOv3 detection algorithm to detect defects by changing the YOLOv3 algorithm's hyperparameters, namely the batch size and input picture size in the NEU-DET dataset. Li et al.^15^ employed the YOLOv4 algorithm for defect identification, incorporating an attention mechanism module in the backbone network of YOLOv4 and modifying the network for route aggregation to a customized block structure for the receptive field. These methods greatly increase the detection rate against the two-stage detection algorithms, but the detection accuracy has not significantly increased. For the purpose of maintain the benefits of quick detection speed and enhance the aforementioned algorithms' detection precision, the YOLOv5^16^ algorithm was proposed and widely studied by many scholars.

Wang et al.^17^ implemented the convolutional block attention module (CBAM) into the YOLOv5 algorithm to enhance the characterization of target features by assigning weights to impair the feature extraction of complicated backdrops. Le et al.^18^ enhanced image features by coordinate attention and fused multiscale features using BiFPN to lower small target sample missed detection and false detection rates. Chen et al.^19^ suggested a more effective detection model based on YOLOv5, employing a new clustering approach to produce anchor frames suitable for PCB defective datasets and a Swin transformer as a feature extraction network, improving detection accuracy and efficiency. Li et al.^20^ have presented a novel two-stage target defect detection approach, which accomplishes localization and classification tasks using two distinct models. Liu et al.^21^ presented using the YOLOv5 algorithm an enhanced YOLO-extract detection model, which combines the hybrid dilation convolution with a newly designed residual structure increase the model's ability to extract shallow features and location information. Due to numerous little defects on the surface of industrial components that closely resemble the backdrop, the original YOLOv5 algorithm is somewhat insufficient in detecting defects and is susceptible to missed and false detection. Therefore, a detection algorithm applicable to the situation of industrial component surface defects that can accurately identify target defects is particularly important. Inspired by the YOLOv5 algorithm, this paper innovates the YOLOv5 algorithm from three aspects and suggests a highly precise detection model for industrial component surface defects. Compared to previous works in surface defect detection in industrial components, our method offers the following contributions.

- The original spatial pyramid pooling structure is innovated by proposing the SPPFKCSPC module, improving the capacity of the algorithm to extract and fuse multiscale features.
- Combining C3 with SPPFKCSPC, named the C3-SPPFK module, and changed the C3 module of the backbone network to C3-SPPFK, improving the network's receptive field and the expression capability of image characteristics.
- The CA is inserted into the neck network of the YOLOv5 algorithm, and the bounding box regression loss function of the algorithm is improved to EIOU, which simultaneously increases the accurate of the model for target recognition and localization, and the whole performance of the network for detection algorithms.

The rest of this essay is structured as follows. "Related work" describes the innovative ideas in this paper. "Methods" addresses the algorithm improvement in detail. "Experiment" describes the experimental design and examines the findings of the experiment. "Conclusion" concludes with conclusions and recommendations for future work.

## Related work

There are various types of surface defects in industrial components. In most cases, they do not differ much in appearance, and the defects are not only mostly similar to the component background but also have tiny defects such as points and lines. This leads to the fact that even if the YOLOv5 algorithm is used for its detection, the accuracy indices obtained from the detection still cannot reach the desired results. Therefore, improving the algorithm's precision becomes a new challenge when detecting targets with the above defects. In this regard, several updated methods built on YOLOv5 have been presented, aiming to increase the algorithm's detection accuracy for complex and diverse defects.

Zhao et al.^22^ presented an improved YOLOv5 lightweight detection technique. While adding a ghost bottleneck to the YOLOv5 detection algorithm, the SENet is also used to the backbone network, and the Conv module in the neck network is substituted deep convolution. Both the parameters for training are reduced and the detection ability of the algorithm is enhanced. Zhang et al.^23^ introduced a WTB defect detection model, which introduced a microscale detecting layer according to the YOLOv5 algorithm and CBAM in every feature fusion layer, greatly reducing the feature information loss of minor target defects and detecting target defects quickly and effectively. Li et al.^24^ proposed a YOLOv5 defect detection algorithm that combines the attention mechanism with receptive fields. The CA and spatial-channel sequeeze excitation (scSE) attention mechanisms are embedded in the algorithm to focus on different features. In addition, a four-channel detection method is used to enlarge the area of detection to realize fast localization and finer processing of small targets. DAI et al.^25^ proposed a simply and efficient model called YOLO-Former, providing a vision transformer to support dynamic attention and global modeling, enhancing the feature representation by using the CBAM module, and we also improved the network structure of the algorithm at the hierarchical level to address the various requirements. TANG et al.^26^ improved YOLOv5 by increasing the detection layer of the algorithm, mitigating the aliasing effect during feature fusion and enhancing feature richness; presenting the adaptively spatial feature fusion (ASFF)spatial feature fusion module and the CA attention mechanism module, greatly increasing the detection accuracy of the algorithm.

When the above improved algorithm is used to detect defects in industrial components, all the evaluation indices are more or less improved relative to the pre-improvement period. However, there is still a substantial promotion space in detection accuracy, and the occurrence of misdetection and leakage phenomena still needs to be reduced.

The method in this paper is more concerned with the enhancement of the image feature extraction and recognition capabilities, enhancing the algorithm's capacity for detection. The suggested approach in this research differs from earlier methods in the following ways.

- The spatial pyramid pooling structure of the algorithm is innovated to strengthen the power of the YOLOv5 algorithm to fuse multiscale features so as to better capture the feature information of the target object at different scales.
- C3 is combined with the innovative spatial pyramid pooling module and replaces the backbone network's C3 module with the newly integrated module. This improves image feature expression, enhancing the algorithm's ability to perform feature extraction on defective images.
- The CA mechanism of attention is integrated into the neck network of the algorithm to increase the algorithm's accuracy in locating and recognizing defective images. The regression loss function of the algorithm is improved as EIOU to enhance the overall network performance to compensate for the increase in training time and slower convergence due to the increase in network structure.

## Methods

### Spatial pyramid pooling

**Figure 1:**
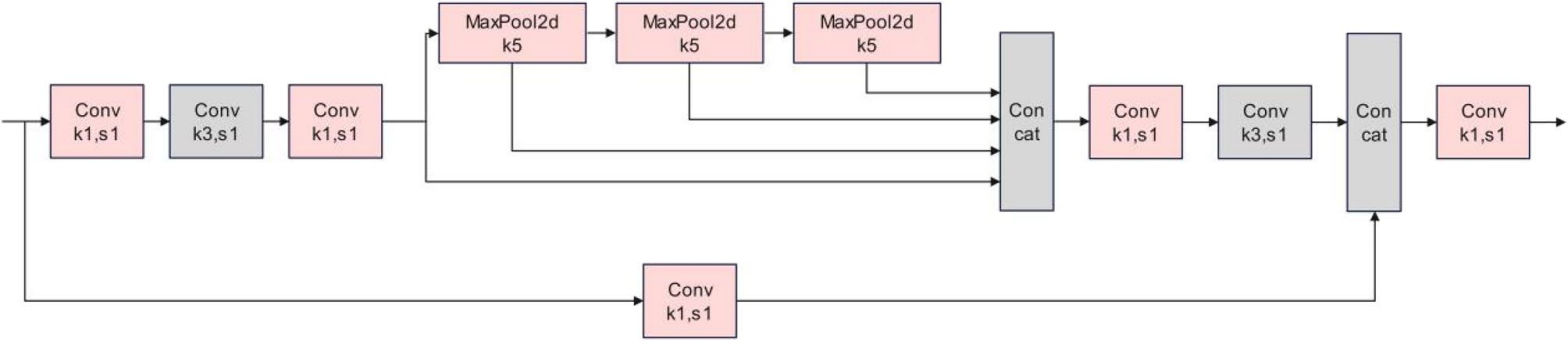
Structure of SPPFCSPC.

**Figure 2:**
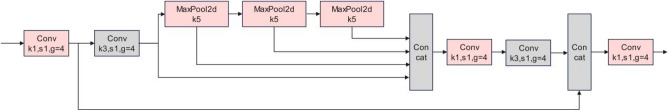
Structure of SPPFKCSPC.

SPP is fully known as the spatial pyramid pooling structure. SPP is proposed to solve two problems: effectively avoid image distortion and other problems brought on by cutting and scaling processes on the image region and overcome the convolutional neural networks issue on the graph related to repetitive feature extraction, greatly improving the generation speed of candidate frames and decreasing computational cost. The role of SPP in the YOLOv5 algorithm is to extract and fuse multiscale features, extract features from different scales and stitch together the feature representations of the same feature map at different scales. It improves the feature map's capacity for expressiveness, which is favorable to the case of large differences in target size to be detected in the image, and greatly improves complex multitarget detection accuracy. Currently, the YOLOv5 algorithm uses the SPPF module, which outperforms SPP. The SPP module divides the input channels in half using a common convolutional module first and then utilizes max pooling with kernel sizes of 5, 9, and 13, concatenates the results of the three max poolings with the data without the pooling operation, and finally, merges them. However, SPPF uses three 5 × 5 max poolings as a substitute, and several small-size pooling kernels cascade instead of a single large-size pooling kernel, thus retaining the original functionality. That is, the running speed is further improved by fusing the feature maps of various receptive fields, which enriches the expressive power of the feature maps. With the continuous innovation of the YOLO algorithm, the module for the spatial pyramid pooling is further improved into SPPCSPC^27^ and SPPFCSPC^28^. The CSP structure is utilized to divide the features into two parts, one of which is processed for regular convolution, and the other is processed for the SPP structure. Finally, these two parts are spliced together, enhancing the feature transfer and information interaction. However, when SPPCSPC and SPPFCSPC are used to modify the YOLOv5 algorithm and validated using the defective dataset, the improvement is not obvious, and the parameters and the calculation become more complex. Through the comparative analysis of the model structure, it is inferred that after the model structure becomes complicated, the training iteration contains many different parameters, resulting in a slow network training convergence speed. Even though the detection accuracy should improve, the detection effect of the YOLOv5 algorithm is not favorable. To obtain better results, the SPPFCSPC module was optimized through continuous experiments, and the improved module was named SPPFKCSPC. The formulas to achieve the improved module are shown in (1), (2) and (3). A comparison with the original structure diagram shows that the improved model reintegrates the model after subtracting two Conv layers. Additionally, the convolution is grouped, and the number of operations and parameters during training is reduced by using group convolution. Experimental verification shows that after improving the SPPF of YOLOv5 to SPPFKCSPC, the mean average precision improves by two percentage points. (1) The structure diagrams of SPPFCSPC and the improved module are shown in Figs. 1 and 2, In Fig. 1, "Conv" refers to the convolution module with the convolution kernel sizes of 1 × 1and 3 × 3, "Maxpool2d" refers to the max pooling module with the size of 5 × 5, and "Concat" refers to the connection module representing dimensional concatenation. The convolution module in Fig. 2 is a grouping operation of convolution based on Fig. 1.

1 $$\begin{array}{*{20}c} {{\text{x}}_{{1 }} = {\text{C}}_{{{\text{k3}}}} {\text{(C}}_{{{\text{k1}}}} {\text{(x))}}} \\ \end{array}$$

2 $$\begin{array}{*{20}c} {{\text{x}}_{{2}} = {\text{ x}}_{{1}} \oplus {\text{M}}\left( {{\text{x}}_{{1}} } \right) \oplus \left[ {{\text{M}}\left( {{\text{M}}\left( {{\text{x}}_{{1}} } \right)} \right)} \right] \oplus \left[ {{\text{M}}\left( {{\text{M}}\left( {{\text{M}}\left( {{\text{x}}_{{1}} } \right)} \right)} \right)} \right]} \\ \end{array}$$

3 $$\begin{array}{*{20}c} {{\text{y }} = {\text{ C}}_{{{\text{k1}}}} \left[ {{\text{C}}_{{{\text{k1}}}} \left( {\text{x}} \right) \oplus {\text{C}}_{{{\text{k3}}}} \left( {{\text{C}}_{{{\text{k1}}}} \left( {{\text{x}}_{{2}} } \right)} \right)} \right]} \\ \end{array}$$

where "x" and "y" stand for the module's inputs and outputs, respectively, "${\text{C}}_{\text{k1}}$" and "${\text{C}}_{\text{k3}}$" denote the convolution kernel sizes of 1 × 1 and 3 × 3, respectively, "⊕" denotes the dimensional splicing operation carried out by the Concat connectivity layer, and "M" denotes the max pooling of Maxpool with a size of 5 × 5.

### C3-SPPFK

**Figure 3:**
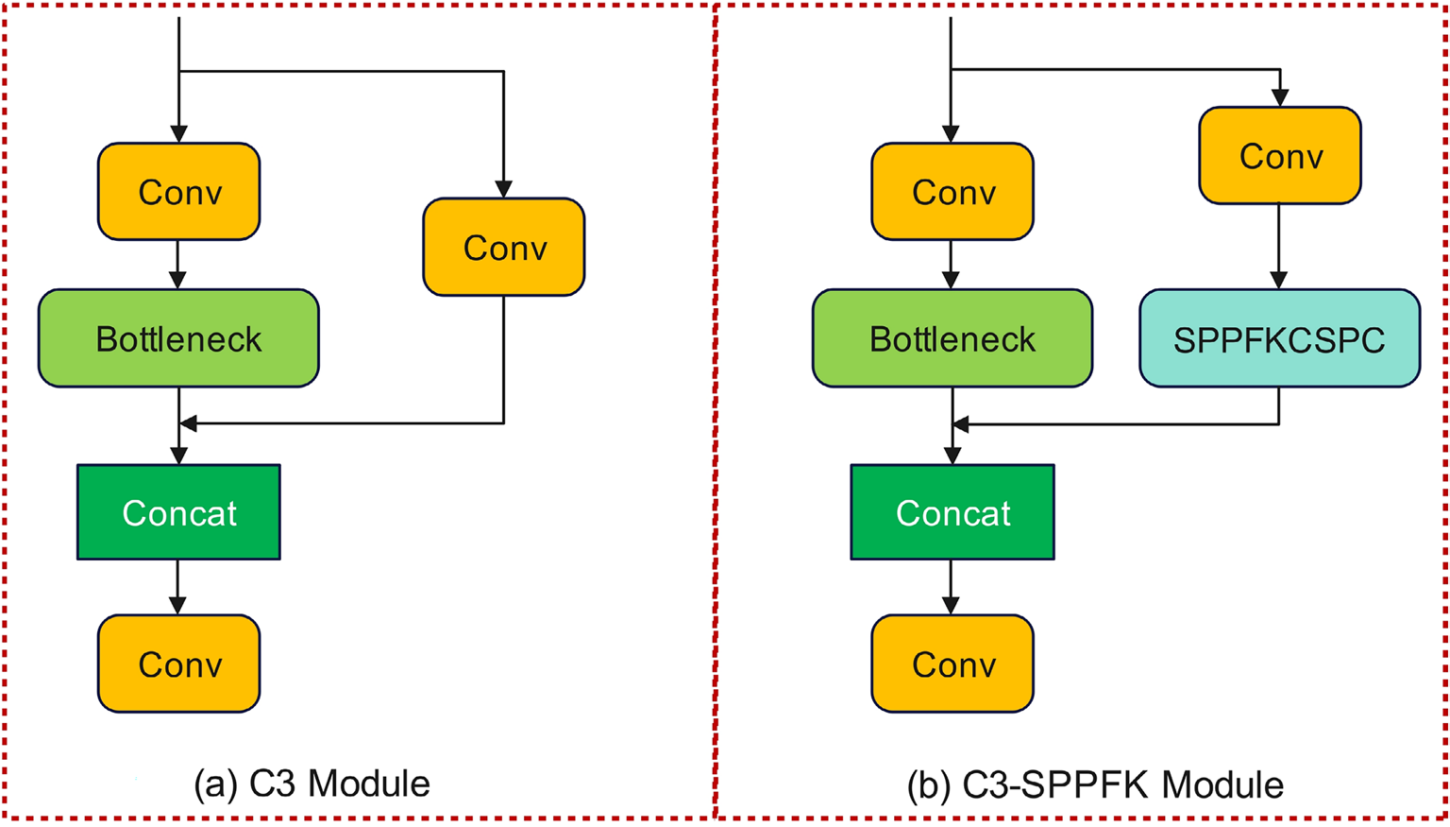
(**a**) Structure of C3 (**b**) structure of C3-SPPFK.

The main role of the C3 module is feature fusion at several feature scales, which enables the network to gather contextual data at various scales. Although the C3 module can extract local features better, it is not sufficient for global features, and the C3 module may cause information loss when processing deep features. To solve the above problems, the C3 module is combined with the SPPFKCSPC module to form a new module (C3-SPPFK), and the structure of C3 and C3-SPPFK as displayed in Fig. 3, (1) in Fig. 3, "Bottleneck" is a module that is composed of two 1 × 1 convolutional layers with a 3 × 3 convolutional layer added in the middle, and "SPPFKCSPC" is a new module proposed after innovation in this paper. By partitioning the input feature map using pooling layers of different sizes and performing pooling operations on each partition, the C3-SPPFK module can extract richer contextual information for better global feature processing. By using the SPPFKCSPC module, C3-SPPFK not only improves feature expressiveness but also increases the network's receptive field, which enables the network to better understand the whole image. Therefore, the C3-SPPFK module has better adaptability and expressiveness compared to the C3 module and performs better in dealing with global feature extraction and avoiding information loss. In this paper, C3-SPPFK replaces the backbone network of YOLOv5 algorithm with C3 module, which improves the representation of target features in the backbone network and decrease the information loss of features.

### CA attention mechanism and regression loss function

**Figure 4:**
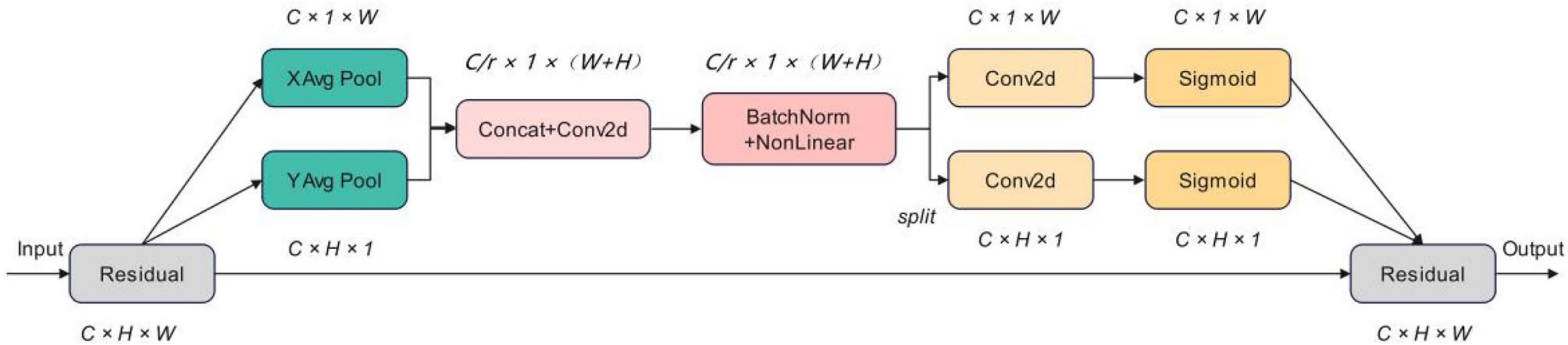
Structure of CA.

**Figure 5:**
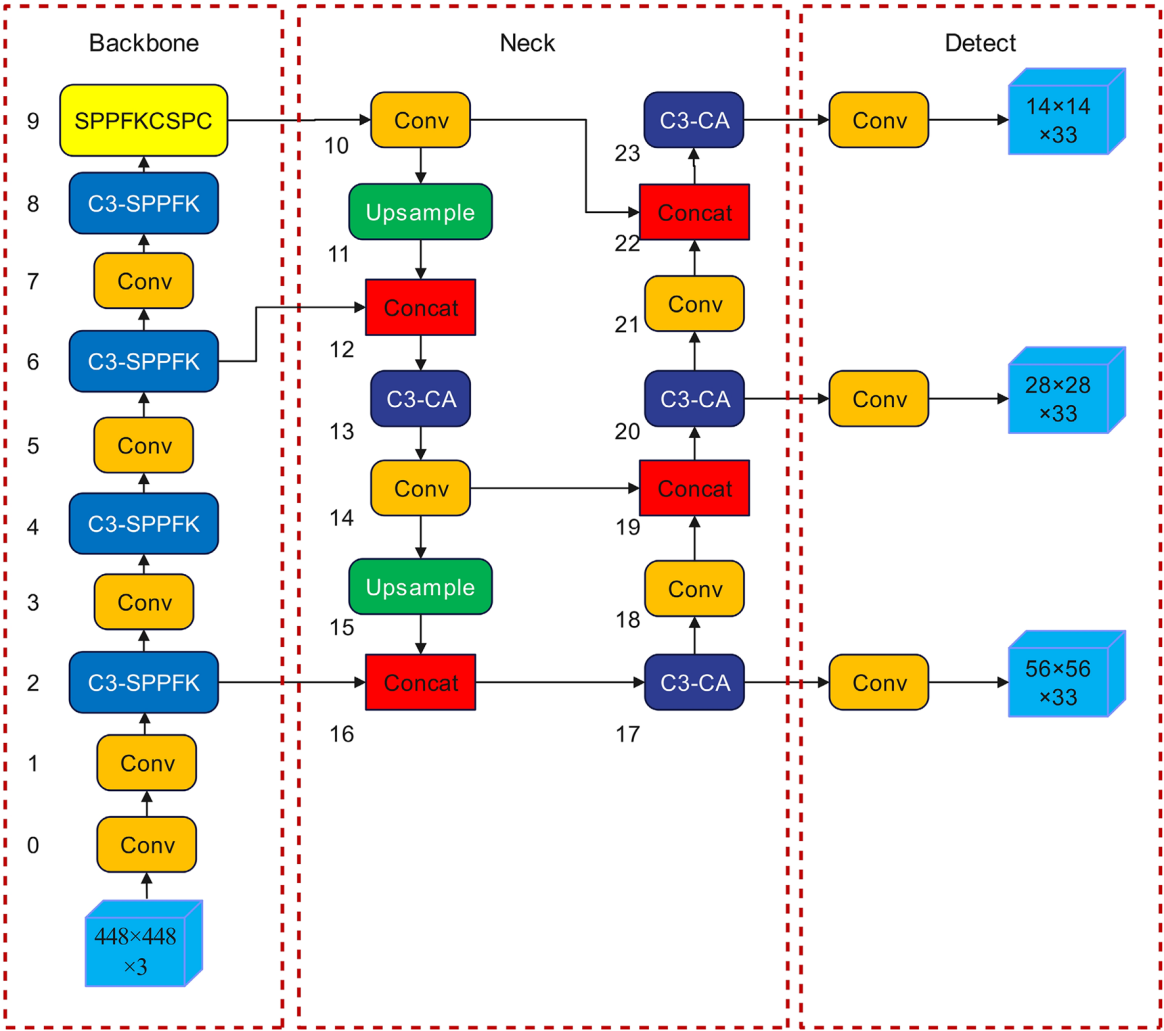
Structure of YOLOv5 model after innovation.

Coordinate attention mechanism (CA)^29^ not only considers the channel information, but also the location information. Therefore, it can easily extract the feature information across the channel, which is why it can easily extract cross-channel feature information to accurately localize and identify the target area. The structural diagram for the model of CA is displayed in Fig. 4, (1) "Residual" in Fig. 4 is the residual structural module, which is conducive to solving the problem of gradient disappearance and gradient explosion. "XAvgpool" and "YAvgpool" are average pooling modules in horizontal direction and vertical direction respectively. "Concat + Conv2d" is a convolution layer and a 1 × 1 convolution layer. "BatchNorm + NonLinear" is a combination of batch standardization and nonlinear regression modules, which is used to encode spatial information in vertical and horizontal directions. "Sigmoid" is the activation function. In this paper, the CA-C3 module^30^ formed by combining the CA with the C3 module is embedded in the neck network of the YOLOv5 algorithm. Figure 5 displays the whole model diagram following the innovation, (1) In Fig. 5, "C3-SPPFK" and "C3-CA" are the new modules formed after the above improvements, "Upsample" is the upsampling module, and "Conv" is the combination of standard convolution, batch standardization, and activation function modules. Multiple convolutional layers are used for extracting multi-scale features in this C3 module. and the CA module can adaptively change the channel importance weights. Paying greater consideration to the features that aid in target classification, which enhances the algorithm's capacity to recognize some of the defects in a similar context; The convolutional layer in the C3 module extracts features at different scales, while the CA module can guide the information exchange between feature maps, which enables semantic features to be combined with detailed features, and also helps to improve the model's detailed representation of the target and semantic understanding.

The original regression loss function CIOU of the YOLOv5 algorithm does not take into account the mismatch between the projected and real frameworks very well. In order to deal with the situation, this research enhances the regression loss function in the YOLOv5 algorithm to EIOU^31^, which considers the IoU between the target boxes and the differences between the centroid and the aspect, Thus, providing a more comprehensive measure of similarity between the projected and real frameworks. EIOU replaced the aspect ratio by calculating the difference in width and height respectively on the original basis which introduced focal loss to overcome the difficulty sample imbalance problem. Improving the regression loss function to EIOU enhances the network's overall performance while compensating for the increased training time and slower convergence caused by increasing the network structure.

## Experiment

To validate the detection effectiveness of the innovative YOLOv5 algorithm, in this paper, we first carried out comparison experiments with SSD, YOLOv5, and some of the improved algorithms based on YOLOv5 using the publicly available NEU-DET and PV-Multi-Defect datasets, and then the NEU-DET dataset was used for ablation experiments.

### Analysis of datasets

**Figure 6:**
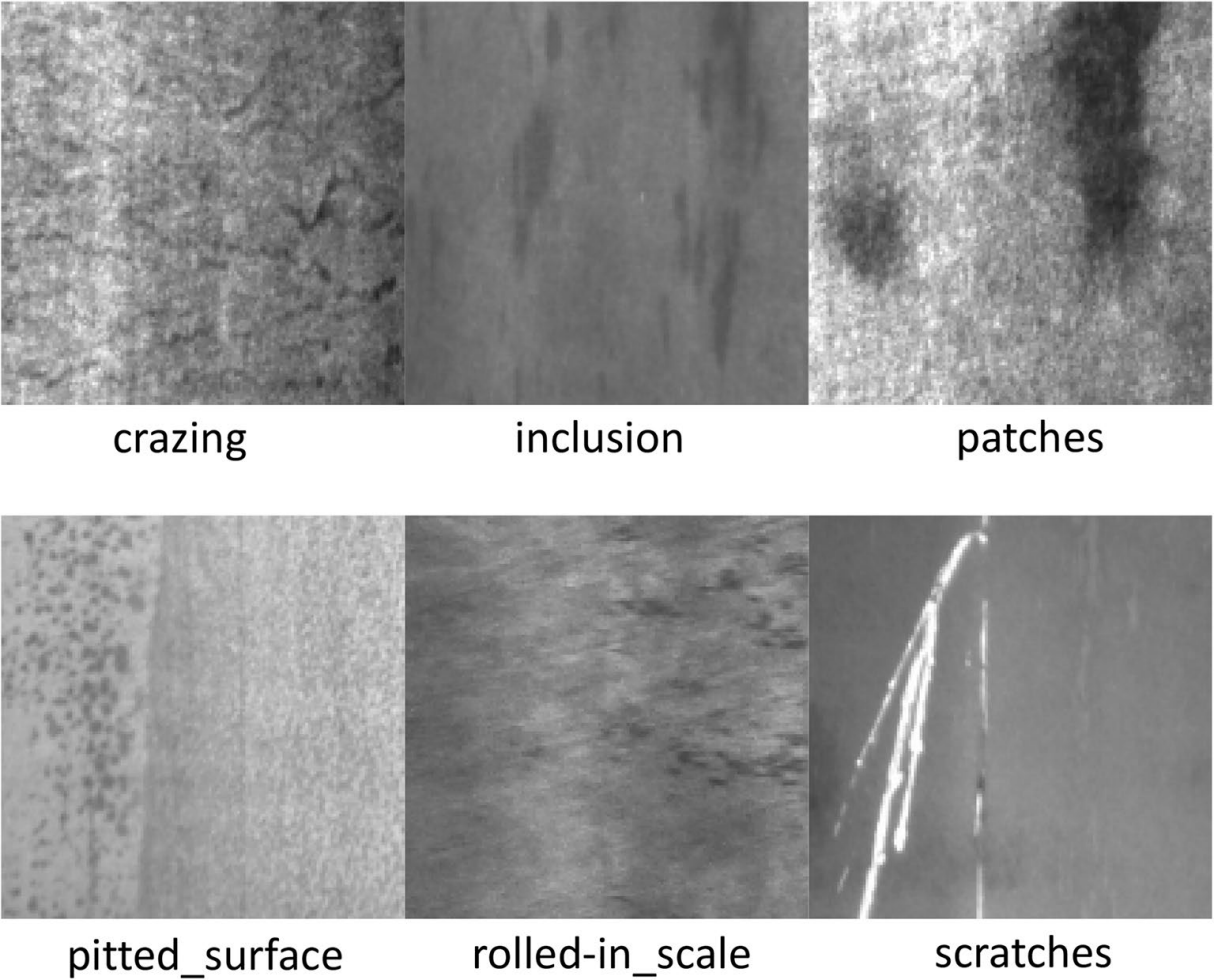
Types of NEU-DET defects.

The public dataset NEU-DET offered by Northeastern College was the one used in this study, which contains 1800 images in all. Among them, each flaw has 300 images, and there are six different sorts of defects. These six defects are "crazing", "inclusion", "patches", "pitted_surface", "rolled-in_scale" and "scratches". The defect sample images are shown in Fig. 6. "Crazing" mainly appears as line defects with crack bars distributed on the steel surface. The "inclusion" defects are roughly nonmetallic inclusions in the form of spots, patches or lines. "Patches" defects are irregular in shape and mostly black and gray in color. "Pitted_surface" defects are generally localized pits and regionally distributed. "Rolled-in_scale" defects are characterized by the presence of black dots or streaks. "Scratch" defects are mainly straight and fine bright lines. These defects are characterized by complex shapes and irregular distributions, increasing the difficulty of detecting them.

**Figure 7:**
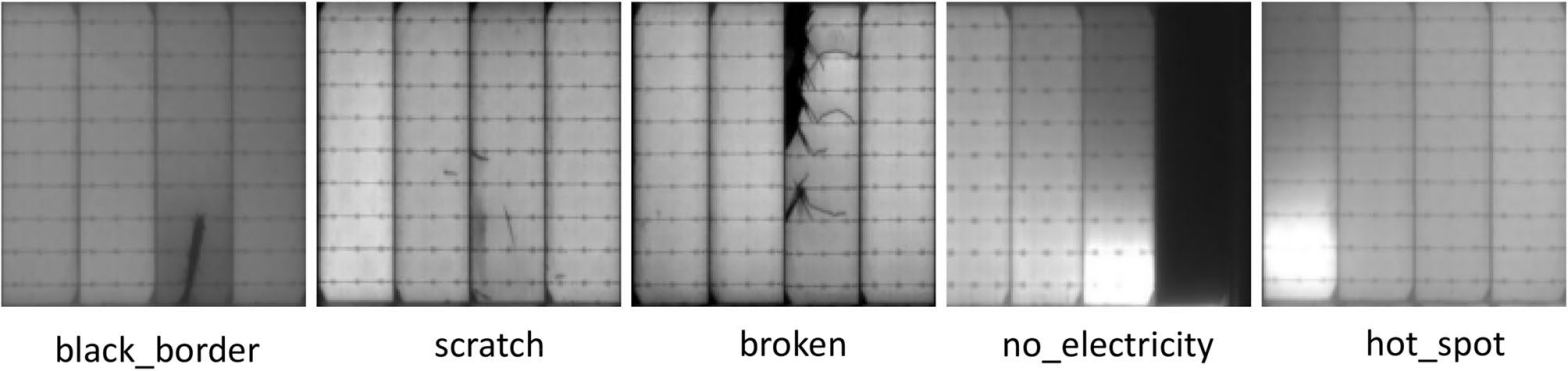
Types of PV-multi-defect defects.

For the purpose of evaluating the accuracy and applicability of the enhanced algorithm, it was examined using the publicly available PV-Multi-Defect dataset. The PV defect detection database contains five types of defect targets and 1100 images, including "black_border", "scratch", "broken", "no_electricity" and "hot_spot". Each image has a different defect distribution, which is more in line with detecting practical applications. The defective sample images are shown in Fig. 7. In our experiments, the two datasets are split into training, testing and validation sets at a proportion of 6:2:2.

### Evaluation indicators

In target identification, the commonly used evaluation indicators are generally precision, recall, P-R curve, F-score, FPS, mAP@0.5 and mAP@0.5:0.95. Precision refers to the proportion of correctly predicted targets among all targets predicted by the model. Recall refers to the proportion of targets that are predicted correctly out of all true targets. The precision-recall curve is produced by charting the precision on the vertical axis, and the recall on the horizontal axis, abbreviated as the P-R curve. The P-R graph visually displays the recall and precision of the learner on the sample population. The F-score provides a more comprehensive evaluation by comprehensively considering the impact of precision and recall. FPS indicates the number of images that can be processed and evaluated per second. The mean average precision represents the average value of each category of AP, while AP is the area enclosed by the precision recall curve and coordinate axis. The specific equations for the F score, mAP@0.5 and mAP@0.5:0.95 are shown in (4), (5), and (6)^27^.

4 $${\text{F{-}score}} = 2 \times \frac{{{\text{Precision}} \times {\text{Recall}}}}{{{\text{Precision}} + {\text{Recall}}}}$$

5 $$\begin{array}{*{20}c} {{\text{AP}} = { }\mathop \smallint \limits_{0}^{1} {\text{p (R) dR}}} \\ \end{array}$$

6 $$\begin{array}{*{20}c} {{\text{mAP }} = \frac{{\mathop \sum \nolimits_{{{\text{i}} = {1}}}^{{\text{N}}} {\text{AP}}_{{\text{i}}} }}{{\text{N}}}} \\ \end{array}$$

where mAP@0.5 represents the average AP of each defect class while its intersection proportion is 0.5, and mAP@0.5:0.95 is the average accuracy at different intersection ratio thresholds.

### Experimentation and setup

The environmental parameters, configuration parameters, and experimental results of the comparison algorithms for each experiment to be conducted in this chapter that are illustrated in the following Tables 1, 2, 3, 4 and 5.

**Table 1:** Introduction of experimental hardware and software parameters.

Name	Parameter
Experimental platform	tesla server
CPU	8-Core
GPU	Tesla V100-PCIE-16 GB
Operating system	Linux system
CUDA	Version 12.1
Deep learning framework	PyTorch 1.12.1
Language	Python 3.9.7

**Table 2:** Settings of NEU-DET training parameter values.

Parameters	The size of input images	Learning rate	Epochs	Batch size
Settings	448 × 448	0.01	200	32

**Table 3:** Settings of PV-multi-defect training parameter values.

Parameters	The size of input images	Learning rate	Epochs	Batch size
Settings	448 × 448	0.01	300	32

**Table 4:** Comparison experiments using NEU-DET.

Dataset	Method	Precision (%)	Recall (%)	F-score	FPS	mAP@0.5 (%)	mAP@0.5:0.95 (%)
NEU-DET	SSD	66.1	67.4	0.68	16	72.5	42.3
	YOLOv5	76.4	73.1	0.75	30	81.1	46.0
	Zhang et al.^22^	74.1	78.5	0.76	–	84.2	–
	Tang et al.^23^	80.4	76.2	0.78	35	86.0	–
	Li et al.^24^	76.6	79.3	–	–	82.4	46.8
	Dai et al.^25^	83.2	80.4	0.82	24	87.4	51.1
	Zhao^26^	78.5	76.8	0.78	33	84.1	47.6
	Ours	81.2	80.6	0.81	25	88.3	52.3

**Table 5:** Comparison experiments using PV-multi-defect.

Dataset	Method	Precision (%)	Recall	F-score	FPS	mAP@0.5 (%)	mAP@0.5:0.95 (%)
PV-multi-defect	SSD	78.4	76.1	0.77	56	81.0	65.1
	YOLOv5	91.5	92.2	0.92	71	96.0	74.7
	Zhang et al.^22^	91.8	91.5	0.92	–	96.7	–
	Tang et al.^23^	92.0	92.1	0.92	78	96.4	–
	Li et al.^24^	92.7	91.3	–	–	96.1	75.4
	DAI et al.^25^	94.6	94.1	0.94	65	97.2	77.8
	Zhao^26^	91.0	93.5	0.92	76	96.5	73.1
	Ours	92.9	95.0	0.94	67	97.5	78.5

The settings of the training parameters have a great impact on the performance of the model, and the parameters in Tables 2 and 3 are the optimal configurations obtained after many experiments, The method used to adjust the parameters in this research experiment was manual parameter tuning. Training with the default settings to establish a performance benchmark before modifications were made helped to find directions for improvement. Based on the training results obtained under the default setting conditions, the result charts (training loss, validation loss, P, R, mAP), PR curves, confusion matrices, training set into images, test results, and dataset statistical images are analyzed, and combined with the settings of the training parameters in the papers of the relevant directions, the parameters of the training are fine-tuned after repeated experiments, so as to obtain the optimal configuration among the many detection results, although FPS has decreased, it still meets the needs of real-time detection. (2) The experimental setup for the comparative algorithms in Tables 4 and 5 is identical to experiments in the previous literatures.

### Comparative experimental results

The results in Tables 4 and 5 clearly show that the improved algorithm in this paper performs better than YOLOv5 and the SSD detection algorithms. Since the YOLOv5 algorithm provides superior detection results compared with that of YOLOv3, YOLOv4 and other one-stage target detection algorithms, the rest of the algorithms are not validated here in the comparative experiments, but some scholars' improved algorithms based on YOLOv5 are compared in the experiments. Compared with the improved algorithms based on YOLOv5 in the table, the algorithms in this paper generally have better evaluation indices when detecting industrial defects, and the mean average precision is likewise the highest, although FPS has decreased, it still meets the needs of real-time detection. The table also shows that this paper's algorithm has an improved detection performance and better applicability when detecting on both datasets.

### Results of ablation experiments

The purpose of ablation experiments is to investigate the contribution of these parts to the model performance by adding or removing the role of a module, i.e., designing a control group, or control variable method. When performing ablation experiments, the other constituent modules of the model need to be fixed, and only the module to be ablated needs to be changed. After each change, the effectiveness of the model on the set of tests needs to be re-evaluated to see how this change affects the overall model performance. Multiple ablation experiments allow for an accurate assessment of the different components to find the elements that have the most influence on the model's performance. On the NEU-DET dataset, we ran ablation experiments to see whether all of the innovations described in this research are successful in improving performance and if there are any interactions between them.

There are four scenarios we use to represent the different innovations to YOLOv5, and the innovation scenarios have been presented in Table 6.

**Table 6:** Schemes of innovations.

Schemes	SPPFKCSPC	C3-SPPFK	C3-CA	EIOU
1	×	×	×	×
2	√	×	×	×
3	√	√	×	×
4	√	√	√	×
5	√	√	√	√

"SPPFKCSPC" refers to replacing the SPPF module in the YOLOv5 algorithm with SPPFKCSPC; "C3-SPPFK" refers to replacing by C3 module in the backbone part of the algorithmic network structure with C3-SPPFK; "C3-CA" refers to replacing the C3 module in the algorithmic necking network with C3-CA; and "EIOU" refers to replacing the algorithm's regression loss function with EIOU.

Scheme 1 is the original YOLOv5 algorithm;

Scheme 2 refers to improving SPPF as the SPPFKCSPC module;

Scheme 3 refers to further improving the C3 module of the backbone network of the algorithm as C3-SPPFK;

Scheme 4 refers to further embedding the CA attention mechanism in the YOLOv5 neck network based on Scheme 3;

Scheme 5 refers to changing the regression loss function to EIOU based on Scheme 4.

Table 7 displays the penultimate examination outcomes for the five schemes, where "a" serves as "crazing", "b" serves as "inclusion", "c" serves as "patches", "d" serves as "pitted_surface", "e" serves as "rolled-in_scale", and "f" serves as "scratches".

**Table 7:** Experimental results of the five programs.

Schemes	Precision (%)	Recall (%)	F-score	mAP@0.5 (%)							mAP@0.5:0.9 (%)
				a	b	c	d	e	f	All	
1	76.4	73.1	0.75	46.8	81.8	97.2	99.5	66.8	94.4	81.1	46.0
2	76.7	74.1	0.75	54.3	83.8	96.5	98.2	74.1	92.0	83.1	47.5
3	75.3	76.6	0.76	79.8	88.5	99.0	95.5	74.8	73.2	85.1	49.8
4	76.7	82.4	0.79	64.4	85.0	97.2	95.5	87.4	92.5	87.7	52.1
5	81.2	80.6	0.81	88.6	87.9	93.2	95.4	69.3	95.3	88.3	52.3

**Figure 8:**
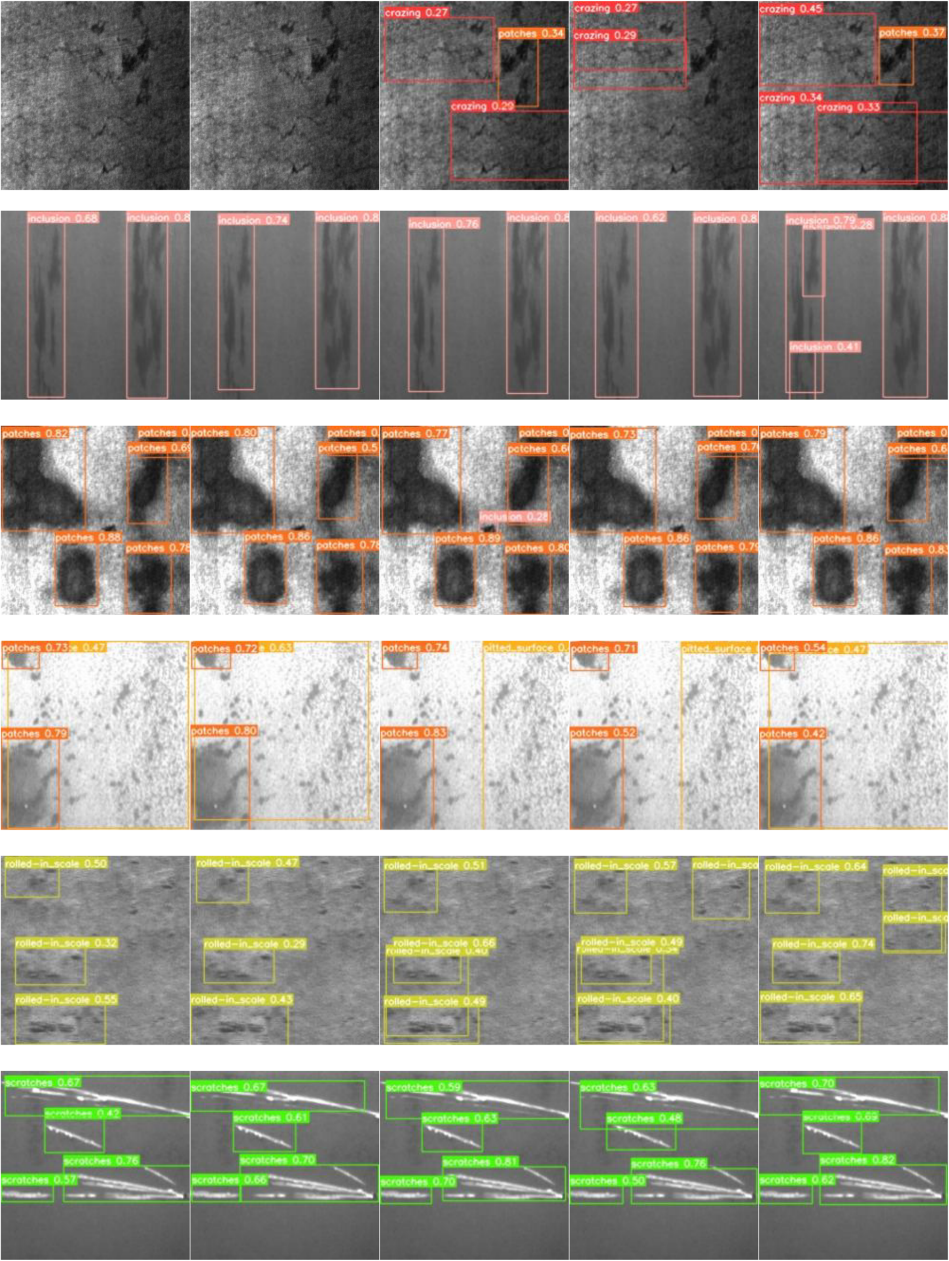
Visualization results for different scenarios.

**Figure 9:**
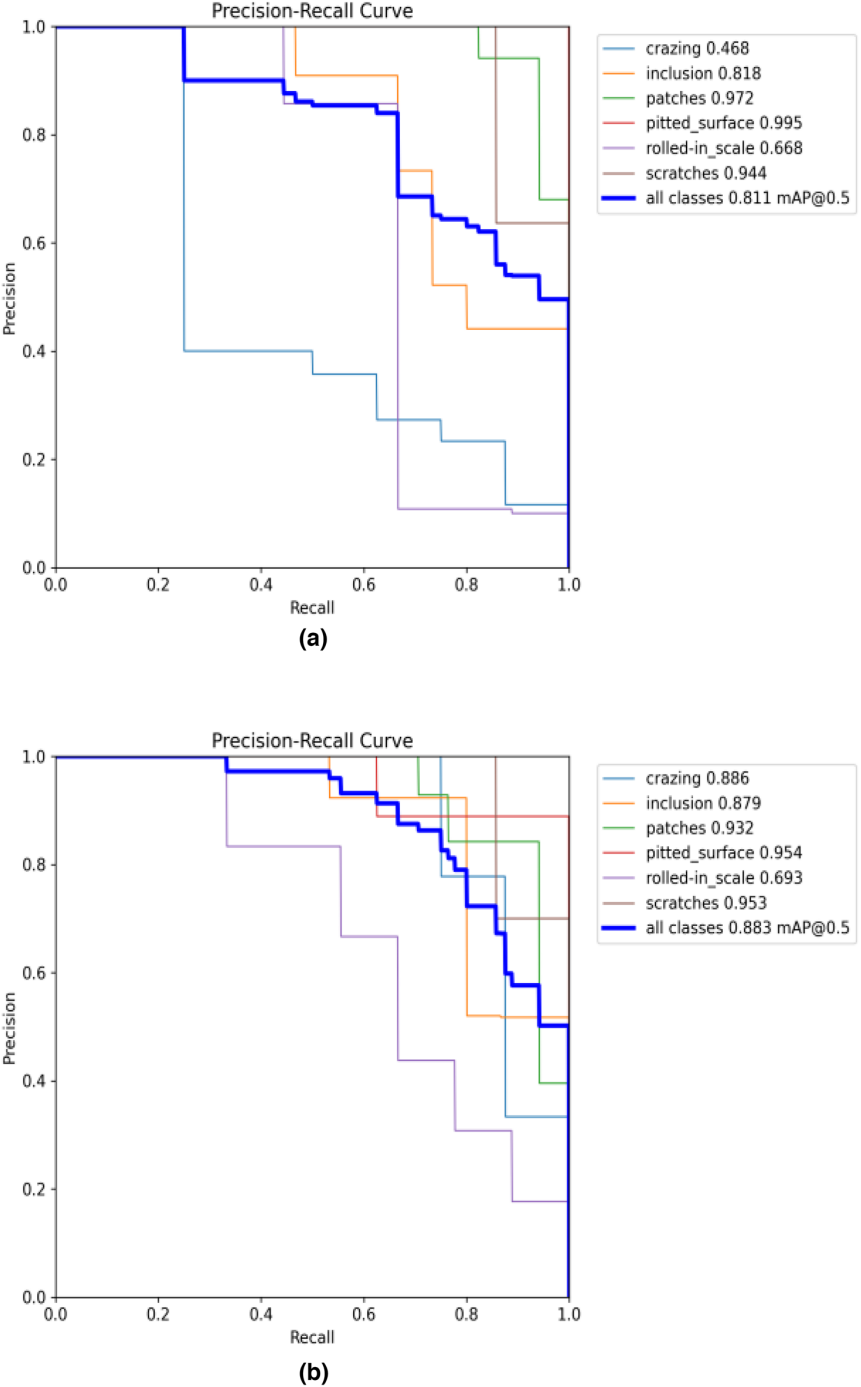
PR curves: (**a**) YOLOv5; (**b**) improved YOLOV5.

The ablation experiment results in Table 7 demonstrate that with the continuous improvement of the YOLOv5 algorithm from Scheme 2 to Scheme 5, the detection effect gradually improved. The mAP values of the six defects in Scheme 5 are almost universally improved, and the rest of the evaluation indices also reach the highest values in comparison to other schemes. All the innovations proposed in this paper achieved positive results. We illustrated several of the detected effects and provided PR curves for verification so as to render the experimental findings in Table 7 more apparent, which are displayed in Figs. 8 and 9, respectively.

Figure 8 shows six different defect types in rows: "crazing", "inclusion", "patches", "pitted_surface", "rolled-in_scale", "scratches", and is divided into five schemes by columns, which are Scheme 1 to Scheme 5. Figure 8 clearly shows that Scheme 1 can only detect defects in the image that are relatively clear and not similar to the background. Some defects were missed, and the accuracy of the detected defects was generally poor. With the step-by-step improvement of the algorithm, it can be seen that more defects were successfully detected from Scheme 1 to Scheme 5. In the six defect images of Scheme 5, it contained practically no missed detections, while the accuracy of detection mostly improved.

The PR curves in Fig. 9 visualize the changes in the average accuracy of the six defects detected before and after the innovation of the algorithm, among which the accuracy of the "crazing" defect most obviously improved, and the total mAP value also greatly improved, from 0.811 to 0.883.

**Figure 10:**
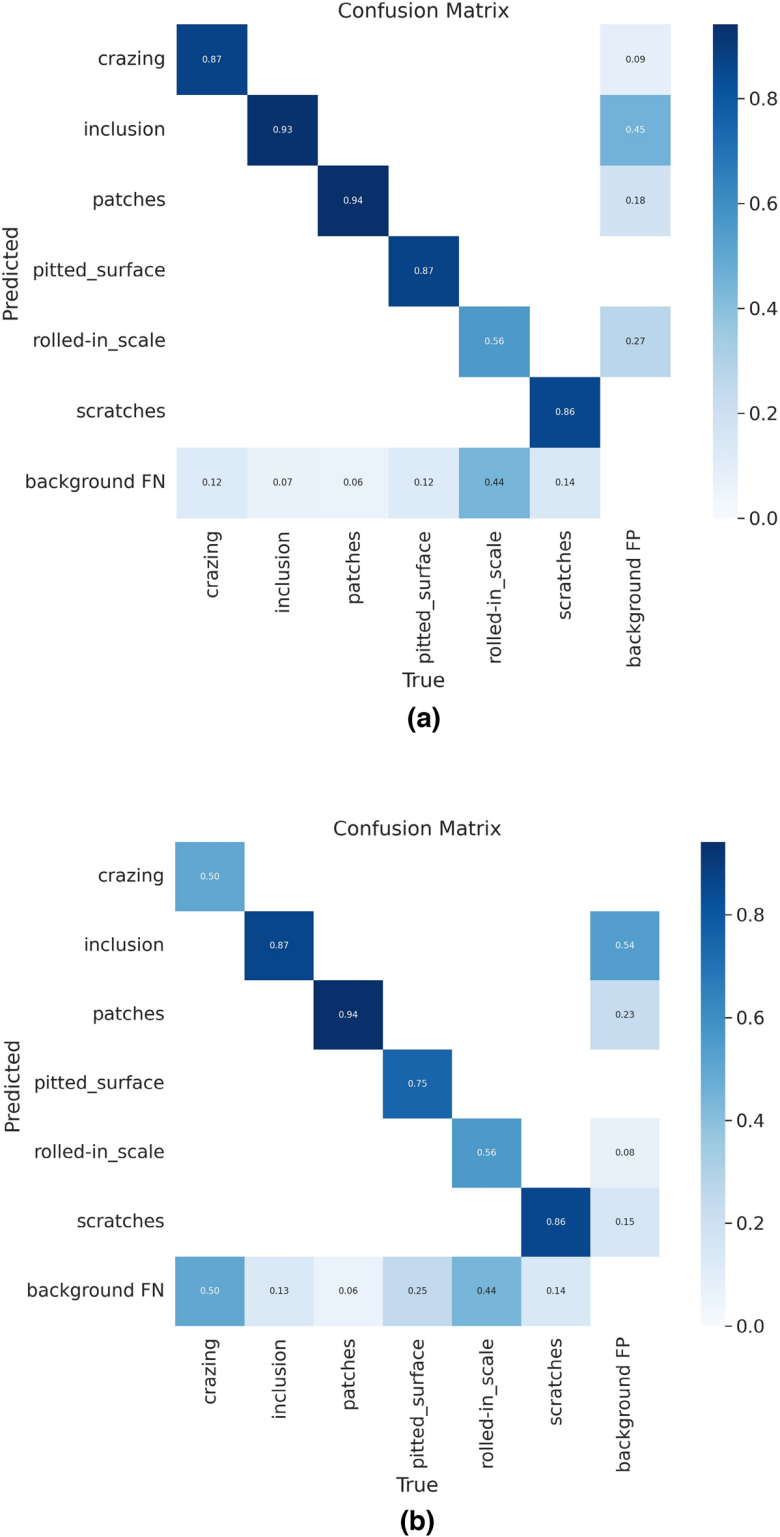
Confusion matrix. (**a**) Improved YOLOv5; (**b**) YOLOV5.

To further investigate the capability of the improvement method to identify various types of defects and the details of defect misjudgment, a multiclassification confusion matrix for quantitatively analyzing the training and testing status of the NEU-DET dataset was introduced. Confusion matrix thoroughly shows the preciseness and error in judgment of real fault kinds in identification. Figure 10 depicts quantized graphs of the matrix of confusion prior to and following the YOLOv5 method was improved. As seen in the graph, the confusion rate of "crazing".

defects was greatly reduced after the improvement, the overall confusion rate between different types of defects was relatively lower, and the new algorithm model was clearly superior to the prior model, which largely reduced the occurrence of false detection.

## Conclusion

This paper takes industrial component surface defects for the purpose of research. To deal with the issue of limited detection accuracy resulting from the existence of industrial component surface defects with complex morphology, irregular distribution and similar background, a new detection model is proposed on the underlying foundation of the YOLOv5 algorithm. The SPPFKCSPC module is suggested to enhance the capacity of multiscale features to fuse. the C3 module of the backbone is replaced by C3-SPPFK to enhancement of the capacity of the algorithm to extract features from defect images. The CA has been embedded in the neck network of the algorithm, and the regression loss function is enhanced to improve the accuracy of the algorithm in identifying and localizing the defect images and enhance the overall network performance. By innovating YOLOv5, the algorithm's capacity to determine and recognize image features was enhanced, thereby improving its detection ability.

The innovative YOLOv5 algorithm allows for the detection of surface defects in industrial components, and through several experiments and tests, it was established that the modified algorithm used in this study improved the overall capability of detecting surface flaws in components. However, the above research shows that there is only a small gap between certain defects, such as the "cracking" and "rolled-in_scale" defects in the NEU-DET dataset, leading to misjudgment between multiple defects during the diagnosis process, resulting in false detection. Therefore, in future research, the recognition accuracy can be further improved by means of targeted data enhancement for different types of defects.

## Data Availability

All data for the experiments used in this study are available via the web. Hyperlinks at the bottom provide links to the datasets. NEU-DET dataset:https://aistudio.baidu.com/aistudio/datasetdetail/195425. PV-Multi-Defect dataset: https://github.com/CCNUZFW/PV-Multi-Defect.
